# Improvements in urinary symptoms, health-related quality of life, and psychosocial distress in the early recovery period after radical cystectomy and urinary diversion in 842 German bladder cancer patients: data from uro-oncological rehabilitation

**DOI:** 10.1007/s00345-024-04839-z

**Published:** 2024-02-29

**Authors:** Henning Bahlburg, Karl Heinrich Tully, Peter Bach, Marius Cristian Butea-Bocu, Moritz Reike, Florian Roghmann, Joachim Noldus, Guido Müller

**Affiliations:** 1 Department of Urology, Marien Hospital Herne, Ruhr-University Bochum, Hölkeskampring 40, 44625 Herne, Germany; 2 Center for Urological Rehabilitation, Kliniken Hartenstein, Bad Wildungen, Germany

**Keywords:** Urinary diversion, Urinary incontinence, Quality of life, Psychosocial distress, Rehabilitation, Radical cystectomy

## Abstract

**Purpose:**

This study aims to investigate urinary symptoms (continence and stoma care), health-related quality of life (HRQoL) and psychosocial distress (PD) in the early postoperative period after radical cystectomy (RC) and urinary diversion for ileal conduit (IC) and ileal neobladder (INB) to obtain a better basis for patient counseling.

**Methods:**

Data for 842 bladder cancer patients, who underwent 3 weeks of inpatient rehabilitation (IR) after RC and urinary diversion (447 IC, 395 INB) between April 2018 and December 2019 were prospectively collected. HRQoL, PD, and urinary symptoms were evaluated by validated questionnaires at the beginning (T1) and the end of IR (T2). In addition, continence status and micturition volume were objectively evaluated in INB patients by 24-h pad test and uroflowmetry, respectively.

**Results:**

Global HRQoL was severely impaired at T1, without significant difference between the two types of urinary diversion. All functioning and symptom scales of HRQoL improved significantly from T1 to T2. In INB patients, all continence parameters improved significantly during IR, while patients with an IC reported fewer problems concerning urostomy management. The proportion of patients suffering from high PD decreased significantly from 50.7 to 34.9%. Age ≤ 59 years was the only independent predictor of high PD. Female patients and patients  ≤ 59 years were more likely to use individual psycho-oncological counseling.

**Conclusion:**

HRQoL, PD and urinary symptoms improved significantly in the early recovery period after RC. Patients with urinary continence reported higher HRQoL and less PD. Psychosocial support should be offered especially to younger patients.

**Supplementary Information:**

The online version contains supplementary material available at 10.1007/s00345-024-04839-z.

## Introduction

Radical cystectomy (RC) has seen an increase of 28% in cases between 2006 and 2019 in Germany. Ileal conduit (IC) and ileal neobladder (INB) are the most common types of urinary diversion [1]. Up to 35% of patients show clinical symptoms of depression after RC and are more likely to commit suicide than the general population [2, 3]. But even before surgery, up to 45% of patients suffer from high psychosocial distress (PD). Advanced tumor stage was associated with high distress, while sex, age, and type of urinary diversion were not identified as predictors of high PD in patients with bladder cancer [4, 5].

In patients with an INB, urinary continence is an independent prognostic factor for a high quality of life (QoL) post-surgery, while incontinence may lead to impaired emotional function [6, 7]. Data comparing QoL in patients with an INB and an IC is conflicting [8–10]. Overall, RC leads to a diminished body image, but patients with an INB are usually more content with their choice of urinary diversion [11]. Health-related QoL (HRQoL) has been identified to predict overall survival in several tumor entities [12, 13].

Supporting patients for reaching the important goal of reintegration into daily life, German social laws entitle cancer patients to an average of three weeks of inpatient rehabilitation (IR). The guideline of the German Society of Urology recommends that all patients be offered several weeks of IR after RC for bladder cancer to minimize functional disorders, reduce PD and improve HRQoL [14]. The design of our study allows us to report on urinary symptoms, QoL and PD in the early postoperative period in a large number of patients after RC in a recent period and in a multi-institutional approach.

## Methods

This prospective study is based on clinical data of patients with urothelial carcinoma of the bladder who underwent RC with creation of IC or INB in various hospitals across Germany and who were treated in a specialized center for urological rehabilitation (Kliniken Hartenstein, Bad Wildungen, Germany) between April 2018 and December 2019. The study protocol was approved by an institutional research committee (research authorization number FF30/2017). At the beginning (T1) and the end (T2) of IR, HRQoL, PD and, urinary symptoms were measured by validated questionnaires (EORTC QLQ-C30, EORTC QLQ-BLM30, QSC-R10, ICIQ-SF; Supplement 1). Furthermore, incontinence and micturition volume in patients after creation of an INB were objectively assessed by 24-h pad test and uroflowmetry, respectively. Social continence was defined as the use ≤ 1 pad per 24 h. Baseline characteristics comprised patients’ age, Karnofsky performance status, body mass index (BMI), the existence of cardiovascular disease and/or diabetes, tumor stage, method of surgery and utilization of neoadjuvant chemotherapy. Since age is known to influence functional outcomes (e.g., urinary continence) and PD, patients were categorized in three age categories (≤ 59; 60–69; ≥ 70 years) [15–17]. Normative data on the HRQoL of the general German population were used for comparison [18].

### Inpatient rehabilitation (IR)

During IR, patients were treated daily by specialized nurses and physiotherapists regarding urinary continence and stoma care, respectively. The multimodal continence therapy includes osteopathic physiotherapy, external urethral sphincter exercises, and educational training on neobladder management and care (e.g., micturition diary with instruction to empty the neobladder initially every 2–3 h during the day as well as at night; careful increase of neobladder volume to achieve sensitivity concerning neobladder volume; and prevention of residual urine volume with special mechanisms for emptying the [19] neobladder). Osteopathic physiotherapy is based on three pillars: (a) visceral techniques to lower intraabdominal pressure and increase arterial circulation and venous and lymphatic drainage, respectively, (b) parietal techniques for myofascial relaxation, and (c) craniosacral techniques aiming at neuronal plasticity resulting in improved function of the external urethral sphincter. Therapy sessions consist of a kinesthetic treatment followed by specific sensorimotor training enabling the patient to differentiate between muscle groups of the pelvis and to isolate the external urethral sphincter. For patients without improvement in daytime continence within 2 weeks of therapy, video-assisted biofeedback-sphincter training via transurethral endoscopy may be performed [15]. Pressure spikes in the reservoir due to increased nightly peristalsis have been identified as one cause of nocturnal urinary incontinence [19]. Patients suffering from severe nocturnal incontinence were, therefore, given anticholinergic drugs at night to reduce neobladder peristalsis [20]. Psychosocial interventions were carried out by physicians, nurses, physiotherapists, psychologists, and social workers. The program includes information on bladder cancer and aftercare, individual, group, and couple psychotherapy, relaxation training, and psychoeducation.

### Statistical analysis

Descriptive statistics for categorical variables included frequencies and proportions, while for continuous variables, medians and interquartile ranges (IQR) or means and standard deviations (SD) were reported. Between-group comparisons were analyzed using the Mann–Whitney *U* test or Chi-square test (Pearson) as appropriate. The Wilcoxon test was used to compare changes in quantitative variables, while the Chi-square test (McNemar) was used to compare changes in proportions. Multivariable logistic regression analyses were performed to identify predictors of high distress or use of individual psychotherapy. Significance was considered at *p* < 0.05. Data were analyzed by SPSS version 29.0 (IBM, Chicago).

## Results

A total of 842 bladder cancer patients underwent IR after RC and urinary diversion (447 IC, 395 INB) in 135 different hospitals in Germany. This being a German cohort, Hautmann INB was chosen in most patients [21]. IR started at a median of 28 days (interquartile range (IQR) 23–35) and ended with a median of 54 days (IQR 48–62) after surgery. The median age was 68 years (IQR 62–75). Men were significantly more likely to receive an INB (52.3 vs 47.7%, *p* < 0.001), while women were far more likely to receive an IC (76.1% vs 23.9%, *p* < 0.001). Patients with an IC were significantly older than patients with an INB (73 years (IQR 67–78) vs 64 years (IQR 58–69); *p* < 0.001). Furthermore, locally advanced disease (≥ pT3; 41.4% vs 24.1%; *p* < 0.001) and lymph node metastases (19.9% vs 11.8%; *p* = 0.002) were significantly more often present in patients with an IC (Table 1).

**Table 1:** Baseline characteristics of 842 patients after radical cystectomy for bladder cancer

Variable	Total	Conduit	Neobladder	p*
Patients, *n* (%)	842 (100.0)	447 (53.1)	395 (46.9)	
Age (years), median (IQR)	68 (62–75)	73 (67–78)	64 (58–69)	** < 0.001**
≤ 59 years, *n* (%)	150 (17.8)	27 (6.0)	123 (31.1)	** < 0.001**
60–69 years, *n* (%)	307 (36.5)	125 (28.0)	182 (46.1)	** < 0.001**
≥ 70 years, *n* (%)	385 (45.7)	295 (66.0)	90 (22.8)	** < 0.001**
Gender, *n* (%)				
Male	683 (81.1)	326 (72.9)	357 (90.4)	** < 0.001**
Female	159 (18.9)	121 (27.1)	38 (9.6)	** < 0.001**
Karnofsky performance status (%), median (IQR)	80 (70–80)	70 (70–80)	80 (70–80)	**0.021**
BMI (kg/m^2^), median (IQR)	25 (23–28)	25 (23–28)	25 (23–27)	0.211
< 30, *n* (%)	743 (88.2)	390 (87.2)	353 (89.4)	0.341
≥ 30, *n* (%)	99 (11.8)	57 (12.8)	42 (11.6)	0.341
CCI ≥ 2, *n* (%)	128 (15.2)	89 (19.9)	39 (9.9)	** < 0.001**
Cardiovascular disease, *n* (%)	547 (65.0)	326 (72.9)	221 (55.9)	** < 0.001**
Diabetes, *n* (%)	114 (13.5)	72 (16.1)	42 (10.6)	**0.021**
Neoadjuvant chemotherapy, *n* (%)	83 (9.9)	37 (8.3)	46 (11.6)	0.102
Method of surgery, *n* (%)				
Robot-assisted cystectomy	93 (11.0)	46 (10.3)	47 (11.9)	0.458
Open cystectomy	749 (89.0)	401 (89.7)	348 (88.1)	0.458
Tumor stage, *n* (%)				
≤ pT2	562 (66.7)	262 (58.6)	300 (75.9)	** < 0.001**
≥ pT3	280 (33.3)	185 (41.4)	95 (24.1)	** < 0.001**
Lymph node positive, *n* (%)**	131 (16.1)	86 (19.9)	45 (11.8)	**0.002**
No. of lymph nodes removed, median (IQR)	17 (12–25)	17 (12–26)	17 (12–24)	0.765

*IQR* interquartile range, *BMI* body mass index, *CCI* Charlson comorbidity index; bold font indicates significant results

^*^Mann–Whitney *U* test or Chi-square test (Pearson) as appropriate

^**^data available for 815 patients (conduit n = 433 and neobladder *n* = 382)

### Urinary continence (Table 2)

**Table 2:** Continence parameters after RC and INB

Variable	T1	T2	p*
Pads during 24-h pad test (*n*), median (IQR)	7 (4–10)	5 (3–8)	** < 0.001**
24-h pad test urine loss (gm), median (IQR)	440 (94–1131)	199 (21–645)	** < 0.001**
Daytime urine loss (gm), median (IQR)	133 (18–596)	27 (0–297)	** < 0.001**
Nighttime urine loss (gm), median (IQR)	248 (44–517)	97 (10–320)	** < 0.001**
Uroflowmetry urine volume (ml), median (IQR)	115 (31–176)	197 (101–290)	** < 0.001**
ICIQ Score, median (IQR)	15 (12–18)	11 (8–15)	** < 0.001**
“Safety pad”, *n* (%)**	69 (18.0)	101 (26.6)	**0.004**
“No pad”, *n* (%)***	7 (1.8)	24 (6.4)	** < 0.001**

*RC* Radical cystectomy, *INB* Ileal neobladder, *T1* Beginning of inpatient rehabilitation, *T2* End of inpatient rehabilitation, *IQR* Interquartile range; bold font indicates significant results

^*^Wilcoxon test

^**^Data available T1: *n* = 383; T2: *n* = 380

^***^Data available T1: n = 383; T2: *n *= 377

Median loss of urine per 24 h decreased by 55% (440 g (IQR 94–1131) vs 199 g (IQR 21–645); *p* < 0.001), while median micturition volume increased by 71% (115 ml (IQR 31–176) vs 197 ml (IQR 101–290); *p* < 0.001) during IR. Median ICIQ-scores significantly decreased during IR (15 (IQR 12–18) vs 11 (IQR 8–15); *p* < 0.001). The percentage of patients with social continence increased from 19.8% at T1 to 33.0% at T2 (*p* < 0.001). At T2, compared to incontinent patients, patients with social continence reported higher global HRQoL (mean 61.2 vs 53.5; *p* < 0.001) and lower PD (median 7 vs 9; *p* = 0.030). Data on continence differentiated by sex can be found in Supplement 2a-b.

### Quality of life

#### EORTC QLQ-C30

Mean global HRQoL was significantly impaired at T1, but improved significantly during IR (39.7 vs 55.8; *p* < 0.001). No significant differences were detected between the two types of urinary diversion at both timepoints. All functioning scales (physical, role, emotional, cognitive and social functioning) improved significantly from T1 to T2 (Table 3). Significant improvements were also detected in all symptom scales and single items except financial difficulties. Patients with an IC were more impacted by fatigue, nausea and vomiting, and constipation at T1 and T2. Meanwhile, patients with an INB suffered significantly more from financial difficulties at both T1 and T2 (Supplement 3).

**Table 3:** QLQ-C30 functioning scales after radical cystectomy and urinary diversion in comparison to normative data [18]

Variable	Normative data (60–69 years)		Total mean (SD)	Conduit mean (SD)	Neobladder mean (SD)	p*
	Male	Female				
Global HRQoL	65.9	65.5				
*T*1			39.7 (21.4)	39.4 (22.0)	39.9 (20.7)	0.656
*T*2			55.8 (19.7)	55.6 (19.3)	56.0 (20.1)	0.872
p**			** < 0.001**	** < 0.001**	** < 0.001**	
Physical functioning	83.0	81.0				
*T*1			59.7 (23.2)	56.7 (23.0)	63.2 (22.9)	** < 0.001**
*T*2			64.3 (21.2)	61.0 (22.0)	68.0 (19.7)	** < 0.001**
p**			** < 0.001**	** < 0.001**	** < 0.001**	
Role functioning	78.6	77.3				
*T*1			35.9 (36.1)	36.9 (36.3)	34.8 (35.9)	0.444
*T*2			48.5 (31.7)	48.1 (31.1)	49.0 (32.4)	0.674
p**			** < 0.001**	** < 0.001**	** < 0.001**	
Emotional functioning	75.7	72.8				
*T*1			54.9 (28.3)	53.7 (28.5)	56.3 (28.0)	0.202
*T*2			70.0 (26.7)	67.4 (27.5)	72.8 (25.6)	**0.006**
p**			** < 0.001**	** < 0.001**	** < 0.001**	
Cognitive functioning	85.1	87.1				
*T*1			74.0 (226.1)	73.0 (26.6)	75.1 (25.5)	0.268
*T*2			81.7 (23.4)	81.1 (24.1)	82.3 (22.6)	0.621
p**			** < 0.001**	** < 0.001**	** < 0.001**	
Social functioning	80.6	83.1				
*T*1			47.9 (32.1)	50.8 (32.8)	44.6 (31.0)	**0.005**
*T*2			62.4 (31.1)	63.8 (31.1)	60.8 (31.0)	0.134
p**			** < 0.001**	** < 0.001**	** < 0.001**	

*QLQ-C30* Quality of Life Questionnaire (cancer patients), *HRQoL* health-related quality of life, *T1* beginning of inpatient rehabilitation, *T2* end of inpatient rehabilitation, *SD* standard deviation; bold font indicates significant results

^*^Mann–Whitney *U* test

^**^Wilcoxon test

#### EORTC QLQ-BLM30

Both micturition problems (61.1 vs 45.3; *p* < 0.001) and urostomy care (38.4 vs 27.9; *p* < 0.001) improved significantly during IR. At T2, 82.8% of patients were autonomously managing their urostomy vs 32.7% at T1 (*p* < 0.001). Future perspective, abdominal bloating/flatulence, and self-esteem/body image all improved significantly in the whole cohort. At T2, patients with an IC were more concerned regarding the future than patients with an INB (47.7 vs 42.8; *p* = 0.043) and suffered more from abdominal bloating and flatulence at both T1 (43.6 vs 36.7; *p* < 0.001) and T2 (31.3 vs 26.3; *p* = 0.003). Results of QLQ-BLM30 domains can be found in Supplement 4.

### Psychosocial distress (QSC-R10)

PD decreased significantly during IR (15 points (IQR 8–23) vs 10 points (IQR 4–18); *p* < 0.001). The proportion of patients suffering from high PD (QSC-R10 ≥ 15 points) decreased significantly from 50.7% at T1 to 34.9% at T2 (*p* < 0.001). Patients with an INB were significantly more likely to receive individual psychological treatment during IR (41.3% vs 34.5%; *p* = 0.042). Results of the QSC-R10 are shown in Supplement 5.

Multivariable logistic regression analysis identified age ≤ 59 years as the only independent predictor for high PD (odds ratio (OR) 2.198; 95% confidence interval (CI) 1.472–3.282; *p* < 0.001) at T1. Age ≤ 59 years (OR 1.964; 95% CI 1.330–2.899; *p* < 0.001) and female sex (OR 1.799; 95% CI 1.236–2.616; *p* = 0.002) were identified as independent predictors for the use of psychological counseling during IR (Table 4). Type of urinary diversion, surgical approach (open vs. robot assisted), neoadjuvant chemotherapy, tumor stage ≥ pT3, and presence of lymph node metastases did neither predict high PD nor use of psychologic counseling during IR.

**Table 4:** Multivariable logistic regression analyses to identify predictors of high distress at the beginning of inpatient rehabilitation (IR) and the use of individual psychotherapy during IR

Variable	High distress (QSC-R10 score ≥ 15)		Use of individual psychotherapy	
	OR (95% CI)	*p*	OR (95% CI)	*p*
Conduit vs. neobladder	0.865 (0.636–1.176)	0.355	1.305 (0.947–1.800)	0.104
Female vs. male	0.969 (0.668–1.405)	0.868	1.799 (1.236–2.616)	**0.002**
Open vs. robot-assisted RC	0.760 (0.486–1.189)	0.230	1.140 (0.715–1.817)	0.582
NAC (yes vs. no)	1.208 (0.742–1.415)	0.448	1.264 (0.779–2.050)	0.343
Age ≤ 59 years vs. ≥ 60 years	2.198 (1.472–3.282)	** < 0.001**	1.964 (1.330–2.899)	** < 0.001**
Tumor stage ≥ pT3 (yes vs. no)	1.025 (0.742–1.415)	0.882	1.064 (0.763–1.485)	0.713
Positive nodal stage (yes vs. no)	1.105 (0.734–1.664)	0.632	1.214 (0.802–1.838)	0.360

*QSC-R10*, questionnaire on stress in cancer patients—10 items*, OR* odds ratio*, CI* confidence interval*, RC* radical cystectomy*, NAC* neoadjuvant chemotherapy; bold font indicates significant results

## Discussion

Our contemporary, prospective study of 842 bladder cancer patients from 135 primary hospitals from all over Germany reveals a moderate to high impairment of HRQoL in the early postoperative period after RC and urinary diversion [18, 22]. These results contradict data from an American single high-volume tertiary center, which did not reveal a strong decrease of HRQoL in a follow-up period of 3 to 24 months after RC and urinary diversion [23]. Fortunately, in our cohort, all functioning and symptom scales of HRQoL improved significantly during IR, but remained diminished nonetheless. Lower HRQoL and impaired physical and emotional functioning in the early postoperative period have been reported by Singer et al. [24]. According to a recent study by Abozaid et al., recovery of both symptom and functioning scales can be expected within 6 and 12 months, respectively [25]. A higher incidence of diarrhea in INB patients and constipation in patients with an IC were also reported by Tyson et al. [26]. A higher burden of financial worries in patients with an INB may sufficiently be explained by their mean younger age. Since a successful return to work after cancer therapy signals successful disease management and may alleviate financial difficulties, employment after cancer therapy is linked to higher HRQoL [27, 28]. Results related to QoL should be interpreted with caution, as the patient cohorts are heterogenous in terms of baseline characteristics. Our cohort mostly underwent open RC (89.0%). Recent data from a prospective randomized single-center trial comparing open RC and robot-assisted RC have shown that HRQoL does not differ between surgical approaches [29]. A bias in our results due to an overrepresentation of patients after more invasive open RC remains possible, but appears to be unlikely.

Urinary continence after the creation of an INB is an important factor for good HRQoL [6, 7]. The assessment of urinary continence after creation of an INB mainly occurs ≥ 1 year after RC and usually includes pad use and incontinence questionnaires [30]. At this point, up to 90% of patients report using ≤ 1 pad/24 h [31]. In our cohort, urinary continence improved significantly during IR. A recent study by our group highlighted the significant influence of younger age and nerve-sparing surgery and the absence of diabetes mellitus or obesity on early continence after creation of an INB [15]. However, female patients still suffer from severe urinary incontinence both at the end of IR, which is consistent with the literature [32]. The importance of urinary continence is further highlighted by patients with social continence reporting both improved HRQoL and lower PD at the end of IR. In this cohort, 27.3% of men and 44.0% of women reported persistent severe urinary incontinence 1 year after RC [33]. Further evaluations, such as urodynamics, could identify further therapeutic options for these patients (e.g., intensified urethral sphincter exercises and biofeedback training).

High perioperative PD in patients undergoing RC has previously been reported by Palapattu et al. [4]. Fear of disease progression has been identified as a major contributor to PD [34]. Worse emotional functioning in patients with an IC in our cohort may thus be explained by a more advanced tumor stage and a higher proportion of lymph node metastases. Psychosocial interventions by a multi-professional rehabilitation team, improving urinary continence, and increasing ability to autonomously care for the urostomy may contribute to significantly decreasing PD in our study. Multivariable logistic regression analysis identified age ≤ 59 years as the only independent predictor for high PD. Age ≤ 59 years and female sex were identified as independent predictors for the use of individual psychological counseling during IR. Female sex as a predictor for psychological counseling during IR is corroborated by studies from Herschbach et al. and Zabora et al. [35, 36]. According to Linden et al., anxiety is highly abundant in younger cancer patients, further supporting our findings [37]. PD should be monitored closely in all patients after RC, but especially so in younger patients at risk, as identified in this study. Referral to self-help groups and psycho-oncologic counseling should be considered if the need arises.

Neoadjuvant chemotherapy (NAC) can significantly prolong survival in patients with muscle-invasive bladder cancer (MIBC) [38, 39] and is recommended by international guidelines [40, 41]. Therefore, it is surprising that NAC was administered in only 9.9% of patients in this cohort. Studies investigating the underlying issues of a widespread administration of NAC in German MIBC-patients are urgently needed.

There are several limitations to our study. As patients from 135 primary hospitals were included in this study, surgical experience may have influenced the choice of urinary diversion and functional outcomes, as reported by Maurice et al. [42]. In our analysis, we did not differ between high-volume and low-volume centers. Furthermore, due to the study design, we could not assess HRQoL prior to RC. IR is specific to the German healthcare system and no control group outside of IR was included in this study. Based on many years of experience in treating patients after RC, it is assumed that almost all patients after RC undergo IR. However, data in this regard are lacking. But the need for close surveillance in the immediate period after RC (e.g., in an IR-setting) is stressed by a recent publication by our group, which showed a complication rate of > 96% in the early recovery period after RC [43].

Overall, this study reports a significant improvement in HRQoL, urinary continence, urostomy management, and PD in a recent cohort of patients in the early postoperative period after RC. Patient-reported outcome measures allow for an individual need assessment, may guide treatment decisions, and should be surveyed regularly during aftercare. Despite its limitations, this study provides important insight into challenges arising during the early postoperative period after RC.

## Conclusion

HRQoL, urinary continence, and PD improved significantly during inpatient rehabilitation after RC and urinary diversion. At the end of IR, patients with social continence reported both better global HRQoL and less PD than patients without social continence. In addition, the percentage of patients able to manage their urostomy independently increased significantly during IR. Psychosocial support should be offered especially to younger patients.

## Supplementary Information

Below is the link to the electronic supplementary material.

Supplementary file1 (DOCX 22 KB)

Supplementary file2 (DOCX 19 KB)

Supplementary file3 (DOCX 21 KB)

Supplementary file4 (DOCX 20 KB)

Supplementary file5 (DOCX 19 KB)

## Data Availability

Data and materials are not publicly available, but are available by the corresponding author upon reasonable request.

## Abbreviations

RC: Radical cystectomy
IC: Ileal conduit
INB: Ileal neobladder
QoL: Quality of life
HRQoL: Health-related quality of life
PD: Psychosocial distress
IR: Inpatient rehabilitation
OR: Odds ratio
CI: Confidence interval
NAC: Neoadjuvant chemotherapy
MIBC: Muscle-invasive bladder cancer
