# Supplementary material for: Improvements in urinary symptoms, health-related quality of life, and psychosocial distress in the early recovery period after radical cystectomy and urinary diversion in 842 German bladder cancer patients: data from uro-oncological rehabilitation
Source: World J Urol. 2024 Feb 29;42(1):111. doi: 10.1007/s00345-024-04839-z (PMC10904548; doi:10.1007/s00345-024-04839-z)
Supplement: Supplementary file 1 — Supplementary file1 (DOCX 22 KB) [file 345_2024_4839_MOESM1_ESM.docx]

**Supplement 1:** Questionnaires

*Quality of Life Questionnaire - EORTC QLQ-C30*

The EORTC QLQ-C30, issued by the European Organization for Research and Treatment of Cancer (EORTC) is a questionnaire specially designed to evaluate QoL in cancer patients [1, 2]. It consists of 30 scored items and includes functioning and symptom scales. A high score in the functional scales equates to a high QoL, while a high score in the symptom scales mirrors a higher burden of symptoms.

*Quality of Life Questionnaire – EORTC QLQ-BLM30*

The QLQ-BLM30 is an addendum to the QLQ-C30 and was developed to evaluate QoL after RC. Disease-specific items such as micturition symptoms or problems concerning the maintenance of the urostomy as well as concerns regarding the future or a negative body image are assessed [3]. Results are interpreted following the QLQ-C30.

*Questionnaire on Stress in Cancer Patients – QSC-R10*

The Questionnaire on Stress in Cancer Patients (QSC-R10) is a standardized and validated 10-item self-assessment instrument [4]. The 10 items cover the most relevant psychosocial aspects of everyday life in cancer patients and are answered on a scale of 0 (“not applicable”) to 5 (“very high burden”). The QSC-R10 total score is calculated by adding up the single items. A sum ≥15 mirrors a high psychosocial burden and should trigger psycho-oncological counseling.

*International Consultation on Incontinence Questionnaire – Short Form (ICIQ – SF)*

The ICIQ-SF is a validated patient-reported assessment and examines frequency and quantity of the involuntary loss of urine and its influence on QoL [5]. The questionnaire consists of three scored items, scored on a Likert-Scale between 0 and 5. Zero points are equivalent to no impairment while a score of 5 points is equivalent to a very high impairment. The total sum of all three scored items allows physicians to classify the patients’ incontinence into three groups. A sum between 1 and 5 points is defined as mild, a score between 6 and 10 points as moderate, and a score ≥ 11 as severe incontinence [6]. Additionally, the number of pads used daily is examined.

[1] Hewitt M, Rowland JH, Yancik R. Cancer survivors in the United States: age, health, and disability. J Gerontol A Biol Sci Med Sci. 2003;58:82-91.

[2] Aaronson NK, Ahmedzai S, Bergman B, Bullinger M, Cull A, Duez NJ, et al. The European Organization for Research and Treatment of Cancer QLQ-C30: a quality-of-life instrument for use in international clinical trials in oncology. J Natl Cancer Inst. 1993;85:365-76.

[3] Danna BJ, Metcalfe MJ, Wood EL, Shah JB. Assessing Symptom Burden in Bladder Cancer: An Overview of Bladder Cancer Specific Health-Related Quality of Life Instruments. Bladder Cancer. 2016;2:329-40.

[4] Book K, Marten-Mittag B, Henrich G, Dinkel A, Scheddel P, Sehlen S, et al. Distress screening in oncology-evaluation of the Questionnaire on Distress in Cancer Patients-short form (QSC-R10) in a German sample. Psychooncology. 2011;20:287-93.

[5] Avery K, Donovan J, Peters TJ, Shaw C, Gotoh M, Abrams P. ICIQ: a brief and robust measure for evaluating the symptoms and impact of urinary incontinence. Neurourol Urodyn. 2004;23:322-30.

[6] Klovning A, Avery K, Sandvik H, Hunskaar S. Comparison of two questionnaires for assessing the severity of urinary incontinence: The ICIQ-UI SF versus the incontinence severity index. Neurourol Urodyn. 2009;28:411-5.
