# Supplementary material for: Improvements in urinary symptoms, health-related quality of life, and psychosocial distress in the early recovery period after radical cystectomy and urinary diversion in 842 German bladder cancer patients: data from uro-oncological rehabilitation
Source: World J Urol. 2024 Feb 29;42(1):111. doi: 10.1007/s00345-024-04839-z (PMC10904548; doi:10.1007/s00345-024-04839-z)
Supplement: Supplementary file 2 — Supplementary file2 (DOCX 19 KB) [file 345_2024_4839_MOESM2_ESM.docx]

**Supplement 2a:** Continence parameters in male patients after RC and INB

| **Variable** | **T1** | **T2** | **p-value*** |
| --- | --- | --- | --- |
| Daytime voiding frequency |  |  |  |
| Median (IQR) | 8 (6–8) | 7 (6–8) | **0.010** |
| Nighttime voiding frequency |  |  |  |
| Median (IQR) | 4 (3–4) | 3 (3–4) | 0.118 |
| Pads at 24-hour pad test |  |  |  |
| Median (IQR) | 7 (4–10) | 5 (3–7) | **<0.001** |
| Pads at day |  |  |  |
| Median (IQR) | 4 (2–6) | 2 (1–4) | **<0.001** |
| Pads at night |  |  |  |
| Median (IQR) | 3 (2–4) | 2 (1–3) | **<0.001** |
| 24-hour pad test urine loss (gm) |  |  |  |
| Median (IQR) | 430 (96–1077) | 184 (21–465) | **<0.001** |
| Daytime urine loss (gm) |  |  |  |
| Median (IQR) | 112 (16–565) | 22 (0–205) | **<0.001** |
| Nighttime urine loss (gm) |  |  |  |
| Median (IQR) | 244 (50–512) | 94 (10–293) | **<0.001** |
| Uroflowmetry urine volume (ml) |  |  |  |
| Median (IQR) | 118 (36–179) | 200 (103–291) | **<0.001** |
| Complete continence**** |  |  |  |
| 24-hour, n (%) | 22 (6.2)** | 66 (19.0)*** | **<0.001** |
| at day, n (%) | 54 (15.3) | 128 (36.8) | **<0.001** |
| at night, n (%) | 36 (10.2) | 81 (23.3) | **<0.001** |
| ICIQ Score |  |  |  |
| Median (IQR) | 15 (12–18) | 11 (8–15) | **<0.001** |

**Abbreviations:**

RC = radical cystectomy

INB = ileal neobladder

T1 = beginning of inpatient rehabilitation

T2 = end of inpatient rehabilitation

IQR = interquartile range

*****Wilcoxon test or Chi square test (McNemar) as appropriate

**indwelling catheter for 3 patients

***indwelling catheter for 9 patients

****Complete continence: no pad use or no urine loss at pad test

**Supplement 2b:** Continence parameters in female patients after RC and INB

| **Variable** | **T1** | **T2** | **p-value*** |
| --- | --- | --- | --- |
| Daytime voiding frequency |  |  |  |
| Median (IQR) | 8 (0–8) | 8 (6–8) | 0.329 |
| Nighttime voiding frequency |  |  |  |
| Median (IQR) | 3 (0–4) | 4 (3–4) | 0.207 |
| Pads at 24-hour pad test |  |  |  |
| Median (IQR) | 7 (4.5–11) | 8 (3.5–11.5) | 0.506 |
| Pads at day |  |  |  |
| Median (IQR) | 5 (3–7) | 5 (2–7) | 0.338 |
| Pads at night |  |  |  |
| Median (IQR) | 3 (1.5–5) | 4 (2–4) | 0.909 |
| 24-hour pad test urine loss (gm) |  |  |  |
| Median (IQR) | 837 (57–1401) | 706 (25–1172) | 0.462 |
| Daytime urine loss (gm) |  |  |  |
| Median (IQR) | 437 (42–690) | 437 (3–716) | 0.249 |
| Nighttime urine loss (gm) |  |  |  |
| Median (IQR) | 251 (40–890) | 279 (15–505) | 0.891 |
| Uroflowmetry urine volume (ml) |  |  |  |
| Median (IQR) | 86 (8–147) | 147 (57–205) | **0.009** |
| Complete continence** |  |  |  |
| 24-hour, n (%) | 6 (15.8) | 7 (18.4) | 1.000 |
| at day, n (%) | 8 (21.1) | 11 (28.9) | 0.250 |
| at night, n (%) | 7 (18.4) | 9 (23.7) | 0.625 |
| ICIQ Score |  |  |  |
| Median (IQR) | 10 (10–15) | 15 (9–18) | 0.437 |

**Abbreviations:**

RC = radical cystectomy

INB = ileal neobladder

T1 = beginning of inpatient rehabilitation

T2 = end of inpatient rehabilitation

IQR = interquartile range

*****Wilcoxon test or Chi square test (McNemar) as appropriate

**Complete continence: no pad use or no urine loss at pad test
