# Supplementary material for: Improvements in urinary symptoms, health-related quality of life, and psychosocial distress in the early recovery period after radical cystectomy and urinary diversion in 842 German bladder cancer patients: data from uro-oncological rehabilitation
Source: World J Urol. 2024 Feb 29;42(1):111. doi: 10.1007/s00345-024-04839-z (PMC10904548; doi:10.1007/s00345-024-04839-z)
Supplement: Supplementary file 3 — Supplementary file3 (DOCX 21 KB) [file 345_2024_4839_MOESM3_ESM.docx]

**Supplement 3:** QLQ-C30 domains after RC – symptoms scales (a) and single items (b)

| **a** | **Total**  mean (SD) | **Conduit**  mean (SD) | **Neobladder**  mean (SD) | **p*** |
| --- | --- | --- | --- | --- |
| Fatigue |  |  |  |  |
| T1 | 61.1 (28.1) | 63.3 (27.7) | 58.7 (28.4) | **0.019** |
| T2 | 45.1 (26.3) | 47.9 (26.9) | 42.0 (25.3) | **0.002** |
| p** | **< 0.001** | **< 0.001** | **< 0.001** |  |
| Nausea and vomiting |  |  |  |  |
| T1 | 14.0 (24.3) | 16.1 (26.1) | 11.7 (21.8) | **0.015** |
| T2 | 6.0 (16.1) | 7.3 (18.2) | 4.6 (13.3) | **0.037** |
| p** | **< 0.001** | **< 0.001** | **< 0.001** |  |
| Pain |  |  |  |  |
| T1 | 33.5 (31.7) | 34.6 (32.4) | 32.2 (30.8) | 0.409 |
| T2 | 23.0 (27.6) | 24.1 (29.0) | 21.8 (25.9) | 0.584 |
| p** | **< 0.001** | **< 0.001** | **< 0.001** |  |

| **b** | **Total**  mean (SD) | **Conduit**  mean (SD) | **Neobladder**  mean (SD) | **p*** |
| --- | --- | --- | --- | --- |
| Dyspnea |  |  |  |  |
| T1 | 35.1 (33.8) | 37.5 (34.1) | 32.4 (33.4) | **0.027** |
| T2 | 25.5 (30.5) | 30.4 (33.0) | 20.2 (26.5) | **< 0.001** |
| p** | **< 0.001** | **< 0.001** | **< 0.001** |  |
| Insomnia |  |  |  |  |
| T1 | 47.3 (37.8) | 47.5 (37.7) | 47.0 (37.9) | 0.855 |
| T2 | 35.9 (34.4) | 37.5 (34.8) | 34.2 (33.9) | 0.191 |
| p** | **< 0.001** | **< 0.001** | **< 0.001** |  |
| Appetite loss |  |  |  |  |
| T1 | 43.7 (37.3) | 45.0 (37.3) | 42.4 (37.2) | 0.318 |
| T2 | 24.6 (31.8) | 27.3 (33.9) | 21.6 (28.9) | **0.049** |
| p** | **< 0.001** | **< 0.001** | **< 0.001** |  |
| Constipation |  |  |  |  |
| T1 | 35.2 (36.3) | 41.5 (37.0) | 28.3 (34.2) | **< 0.001** |
| T2 | 25.4 (32.4) | 30.5 (34.4) | 19.8 (29.0) | **< 0.001** |
| p** | **< 0.001** | **< 0.001** | **< 0.001** |  |
| Diarrhea |  |  |  |  |
| T1 | 25.3 (32.4) | 22.0 (31.9) | 28.9 (32.5) | **< 0.001** |
| T2 | 16.6 (27.5) | 15.1 (26.8) | 18.2 (28.1) | 0.064 |
| p** | **< 0.001** | **< 0.001** | **< 0.001** |  |
| Financial difficulties |  |  |  |  |
| T1 | 22.1 (30.3) | 17.6 (28.1) | 27.3 (31.9) | **< 0.001** |
| T2 | 20.7 (30.1) | 17.3 (28.3) | 24.5 (31.6) | **0.001** |
| p** | 0.189 | 0.648 | **0.019** |  |

**Abbreviations:**

QLQ-C30 = Quality of Life Questionnaire (cancer patients)

RC = radical cystectomy

T1 = beginning of inpatient rehabilitation

T2 = end of inpatient rehabilitation

SD = standard deviation

*****Mann-Whitney-U test

**Wilcoxon-test
