# Supplementary material for: Improvements in urinary symptoms, health-related quality of life, and psychosocial distress in the early recovery period after radical cystectomy and urinary diversion in 842 German bladder cancer patients: data from uro-oncological rehabilitation
Source: World J Urol. 2024 Feb 29;42(1):111. doi: 10.1007/s00345-024-04839-z (PMC10904548; doi:10.1007/s00345-024-04839-z)
Supplement: Supplementary file 4 — Supplementary file4 (DOCX 20 KB) [file 345_2024_4839_MOESM4_ESM.docx]

**Supplement 4:** QLQ-BLM30 domains after RC – conduit versus neobladder

| **Variable** | **Total**  mean (SD) | **Conduit**  mean (SD) | **Neobladder**  mean (SD) | **p*** |
| --- | --- | --- | --- | --- |
| Urinary symptoms |  |  |  |  |
| T1 |  |  | 61.1 (19.9) |  |
| T2 |  |  | 45.3 (19.7) |  |
| p** |  |  | **< 0.001** |  |
| Urostomy problems |  |  |  |  |
| T1 |  | 38.4 (22.0) |  |  |
| T2 |  | 27.9 (21.3) |  |  |
| p** |  | **< 0.001** |  |  |
| Future perspective |  |  |  |  |
| T1 | 58.3 (31.7) | 59.0 (31.5) | 57.5 (32.0) | 0.503 |
| T2 | 45.4 (30.9) | 47.7 (31.6) | 42.8 (29.9) | **0.043** |
| p** | **< 0.001** | **< 0.001** | **< 0.001** |  |
| Abdominal bloating / flatulence |  |  |  |  |
| T1 | 40.3 (29.6) | 43.6 (30.5) | 36.7 (28.2) | **0.001** |
| T2 | 28.9 (26.1) | 31.3 (26.2) | 26.3 (25.8) | **0.003** |
| p** | **< 0.001** | **< 0.001** | **< 0.001** |  |
| Self-esteem / body image |  |  |  |  |
| T1 | 38.0 (31.1) | 39.5 (32.4) | 36.4 (29.7) | 0.275 |
| T2 | 30.9 (29.9) | 32.1 (31.3) | 29.4 (28.3) | 0.498 |
| p** | **< 0.001** | **< 0.001** | **< 0.001** |  |

**Abbreviations:**

QLQ-BLM30 = Quality of Life Questionnaire (after RC)

RC = radical cystectomy

T1 = beginning of inpatient rehabilitation

T2 = end of inpatient rehabilitation

SD = standard deviation

*Mann-Whitney-U test

**Wilcoxon-test
