# Supplementary material for: Improvements in urinary symptoms, health-related quality of life, and psychosocial distress in the early recovery period after radical cystectomy and urinary diversion in 842 German bladder cancer patients: data from uro-oncological rehabilitation
Source: World J Urol. 2024 Feb 29;42(1):111. doi: 10.1007/s00345-024-04839-z (PMC10904548; doi:10.1007/s00345-024-04839-z)
Supplement: Supplementary file 5 — Supplementary file5 (DOCX 19 KB) [file 345_2024_4839_MOESM5_ESM.docx]

**Supplement 5:** Psychosocial distress (QSC-R10) after RC – conduit versus neobladder

| **Variable** | **Total** | **Conduit** | **Neobladder** | **p*** |
| --- | --- | --- | --- | --- |
| Total score |  |  |  |  |
| T1, Median (IQR) | 15 (8–23) | 15 (8–25) | 15 (9–22) | 0.626 |
| T2, Median (IQR) | 10 (4–18) | 11 (5–18) | 8 (4–17) | **0.008** |
| p** | **< 0.001** | **< 0.001** | **< 0.001** |  |
|  |  |  |  |  |
| Cut-off ≥ 15 |  |  |  |  |
| T1, n (%) | 422 (50.7) | 222 (50.7) | 200 (50.8) | 0.982 |
| T2, n (%) | 290 (34.9) | 168 (38.2) | 122 (31.2) | **0.035** |
| p** | **< 0.001** | **< 0.001** | **< 0.001** |  |
|  |  |  |  |  |
| Individual psychological counseling |  |  |  |  |
| n (%) | 317 (37.6) | 154 (34.5) | 163 (41.3) | **0.042** |

**Abbreviations:**

QSC-R10 = questionnaire on stress in cancer patients (10 items)

RC = radical cystectomy

T1 = beginning of inpatient rehabilitation

(data available for 832 patients (conduit n=438 and neobladder n=394))

T2 = end of inpatient rehabilitation

(data available for 831 patients (conduit n= 440 and neobladder n=391))

IQR = interquartile range

*Mann-Whitney-U test, or Chi-square test (Pearson) as appropriate

**Wilcoxon-test or Chi-square test (McNemar) as appropriate
